# A heterogeneous landscape does not guarantee high crop pollination

**DOI:** 10.1098/rspb.2016.1472

**Published:** 2016-09-14

**Authors:** Ulrika Samnegård, Peter A. Hambäck, Debissa Lemessa, Sileshi Nemomissa, Kristoffer Hylander

**Affiliations:** 1 Department of Ecology, Environment and Plant Sciences, Stockholm University, 106 91 Stockholm, Sweden; 2 Department of Biology, Lund University, Sölvegatan 37, 223 62 Lund, Sweden; 3 Ethiopian Biodiversity Institute, Forest and Range Land Plant Biodiversity Directorate, PO Box 30726, Addis Ababa, Ethiopia; 4 Department of Plant Biology and Biodiversity Management, Addis Ababa University, PO Box 3434, Addis Ababa, Ethiopia

**Keywords:** *Brassica napus*, forest cover, landscape composition, pollination services, sub-Saharan Africa, yield gaps

## Abstract

The expansion of pollinator-dependent crops, especially in the developing world, together with reports of worldwide pollinator declines, raises concern of possible yield gaps. Farmers directly reliant on pollination services for food supply often live in regions where our knowledge of pollination services is poor. In a manipulative experiment replicated at 23 sites across an Ethiopian agricultural landscape, we found poor pollination services and severe pollen limitation in a common oil crop. With supplementary pollination, the yield increased on average by 91%. Despite the heterogeneous agricultural matrix, we found a low bee abundance, which may explain poor pollination services. The variation in pollen limitation was unrelated to surrounding forest cover, local bee richness and bee abundance. While practices that commonly increase pollinators (restricted pesticide use, flower strips) are an integral part of the landscape, these elements are apparently insufficient. Management to increase pollination services is therefore in need of urgent investigation.

## 1. Introduction

Global agriculture has become increasingly pollinator-dependent with a disproportionate increase in the area cultivated with pollinator-dependent crops [1]. This trend is more pronounced in the developing than in the developed world [1]. At the same time, bees, the most efficient pollinators, are reported to have declined in many areas of the world, which has raised concerns about increasing yield gaps and decreasing stability of food production [2–4]. These concerns have to some extent been verified; pollen limitation in pollinator-dependent crops has been found to hinder the expected increase in yield and to decrease temporal stability of global production [5]. Sufficient pollination services to pollination-dependent crops are not only of significant economic value [6]; these crops also contribute with essential micronutrients important for human health and increase the overall diversity of human diets [7]. Many human societies that directly rely on pollination services for local food supply and income generation live in geographical regions, where the knowledge about pollinators and the pollination systems is limited [8]. It is therefore vital to explore if the local pollinator community can support the expansion of pollinator-dependent crops, especially in developing countries. Here, we address this gap by evaluating the pollen limitation and pollination success of a common oil crop with a manipulative experiment across a heterogeneous agricultural landscape in southwestern Ethiopia.

The decline in bee populations is often linked to one or a combination of the following stressors: land-use change and intensification, pesticide application, climate change, the spread of pests and pathogens and introduced alien species [2,9]. Higher species richness of pollinators and visitation rates to crops, which leads to correspondingly higher fruit set and stability in fruit set, are often associated with proximity to undisturbed or semi-natural florally diverse vegetation [10,11]. However, despite these general findings, the responses of bees to various human impacts are sometimes ambiguous and may depend on context, besides that different pollinator species and communities differ in their inherent vulnerability [12–16]. Given strong regional differences, it has been argued that the models that have been developed to understand the responses of bees to human impacts cannot be generalized outside the range of included data [17]. Given our very limited knowledge about pollinators in sub-Saharan Africa and the uncertainty of models on bee responses, we need targeted research to establish adequate knowledge on the sub-Saharan pollinators and associated pollination services [18].

In southwestern Ethiopia, landscapes are characterized by heterogeneous, traditionally managed, agricultural land interspersed with various types of forests. Here the external input of agro-chemicals is low and agricultural crops are grown in different landscape settings ranging from surroundings with low forest cover to places that are almost enclosed by forest [19]. The agriculture is small-scale subsistence farming, and crops for household consumption are grown in fields or in the compound of individual households, the homegarden. Smallholder farmers are strongly reliant on their crops and yields both for food diversity and cash income. Therefore, they are also clearly dependent on functioning ecosystems services and thus vulnerable to possible degradation of those. Given the mainly organic management, the diversified fields and the presence of high-quality habitats, we would expect wild pollinators to be abundant and pollen limitation to be low in these landscapes [16,20]. However, in a recent study from this area, we found low abundances of bees (collected with pan and vane traps), both in the end of the rainy season when most herbs and annual crops are flowering as well as in the dry season when most trees are flowering [21]. In the same landscape, but in shaded coffee plantations, semi-wild honeybees were abundant but very few wild pollinators were found visiting flowering coffee at the end of the dry season [22]. In both of these studies, we found that wild pollinators and bee species richness responded positively to forest habitats (more complex forest structures or more forest in the surrounding), but also that wild bees were found in surprisingly low numbers [21,22].

Based on previous findings, our aims with this study were to investigate whether the low pollinator abundance leads to pollen limitation of crops, and if the pollination success varies with the amount of forest cover in the surrounding area. In correspondence to earlier findings for bee abundance and richness, we hypothesized that the pollination success, and thereby relative yield, would be higher in sites with more forested surroundings compared with sites with less forested surroundings. To test this hypothesis, we sowed rapeseed, *Brassica napus* L., which is known to be partly pollinator-dependent, in 23 sites that varied in the amount of surrounding forest cover. We applied three pollination treatments: *control, pollinator exclusion* and *pollen addition*, allowing us to evaluate both the amount of pollination services provided (difference between pollinator exclusion and control plants) and how the pollen limitation (difference between controls and pollen addition plants) varies across the landscape. Finally, we analysed the pollen limitation of rapeseed in relation to bee abundance and richness that we sampled with vane and pan traps in the same homegardens.

## 2. Material and methods

### (a) Study area

**Figure 1.**
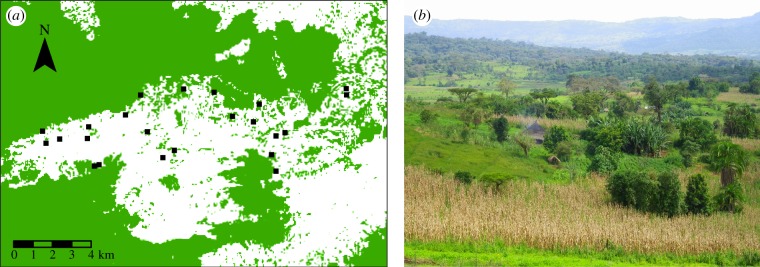
(*a*) The study area in southwest Ethiopia. The black squares show the location of the 23 homegardens where we sowed rapeseed and collected bees with vane and pan traps. The darker areas represent forested areas and the white areas are open land such as agricultural fields, grasslands and wetlands. (*b*) A photo from the study landscape with common structures like homegardens, smaller agricultural fields, scattered trees and grasslands, with larger continuous forest in the background (Photo: U. Samnegård). (Online version in colour.)

We conducted the study in Gera Woreda which is located in Jimma zone in southwestern Ethiopia (7°44′–7°48′ N and 36°18′–36°28′ E) at altitudes between 1900 and 2100 m above sea level (a.s.l.). The annual precipitation varies between 1480 and 2150 mm with most rainfall between June and September (rainy season) and the mean daily minimum and maximum temperature is 12°C and 28°C (data from Agaro's Metrological Station 1996–2012, Ethiopian National Meteorological Service Agency, unpublished document). The landscape is heterogeneous and comprised large remnants of moist Afromontane forests, annual crop fields, grazing land, wetlands and homegardens (figure 1). Homegardens are the compounds of individual households in which the family often grows vegetables, root crops, spices, fruit trees, coffee and other stimulant plants and has livestock rearing [23]. Within the agricultural landscape, the density of trees varies and can sometimes be high, for example, in woodlots of eucalyptus, shaded coffee stands, in live fences and in homegardens. In the crop fields, wind-pollinated cereals such as maize and teff are dominating, whereas pollinator-dependent crops and fruit trees like avocado, guava, peach tree, coffee, pumpkin, field beans and rapeseed are common in homegardens [23,24]. Rapeseed is not grown in the crop fields but is found in smaller plots in approximately 50% of the homegardens and is mainly grown for its oil and fresh leaves [23]. Two species of rapeseed are commonly grown in homegardens, *B. napus* and the local species *Brassica carinata* [25]. It is difficult to get detailed knowledge about the usage of pesticides in the landscape. The herbicide 2, 4-Dichlorophenoxyacetic acid (2, 4-D) is commonly used in crop fields to kill off broadleaf weeds (local agricultural development office, D. Lemessa 2012, personal communication) and sometimes herbicides are used in coffee plantations [22]. However, the general notion is that the usage of insecticides is low in homegardens, crop fields and in semi-forest coffee systems across the landscape (local agricultural development office, D. Lemessa 2012, personal communication) [22,26,27]. Yet, it should be noted that the control is poor regarding the usage of pesticides in Ethiopia and a study analysing the levels of organochlorines in human and cow milk in a nearby larger city found comparatively high levels of DDT and its metabolites; probably coming from indoor spraying against vectors of malaria [28].

### (b) Study design

Homegarden sites were preselected using satellite images in Google Earth (accessed 13 January 2012) and the final selection was done after a ground survey which included an agreement with the homegarden owners [19,21]. The sites were distributed over the landscape and selected to cover a gradient from low to high amount of forest cover in the surroundings (table 1 and figure 1*a*). All sites were situated within a 5 × 17 km area with 250 m as the shortest distance between two sites (figure 1*a*). We started out with 28 sites, but owing to crop failure and damage by livestock we could only collect data from 23 sites.

**Table 1.** Mean, minimum and maximum per cent coverage of the measured landscape parameters surrounding the 23 homegardens at three scales (700 m radius, 200 m radius and in the hectare plot), as well as the mean, minimum and maximum of floral and bee diversity and abundance.

landscape and local variables	mean	min	max
700 m radius			
amount forest (%)	16.9	1.2	36.5
amount wooded areas (%)	37.9	13.2	61.1
200 m radius			
amount forest (%)	13.7	0.1	30.4
amount wooded areas (%)	36.9	8.5	64.9
100 × 100 m			
wooded land including shaded coffee (%)	16.5	0.0	59.3
shrubs including life fences (%)	7.2	0.0	24.4
grazing land (%)	16.9	0.0	67.2
perennial crops (e.g. avocado, banana, coffee, enset) (%)	13.0	0.0	69.7
annual crops, vegetables and chat (%)	39.2	0.0	86.7
floral diversity (number of species)	29	21	42
total floral abundance (number of individual floral units)	57 083	16 800	130 250
total bee diversity (number of species)	10.4	2	20
total bee abundance (number of individuals)	22.1	2	49

In each site, we rented a 5 × 5 m plot of land. Five-eighths of the plots were sown with the local variety of *B. napus*, which is commonly used in southwestern Ethiopia (purchased by us on the market in the town of Agaro)*.* The same type of seeds was sown across all sites and was sown between 27 June and 5 July 2012. We used the local recommended seed sowing density of 12 kg ha^−1^. Before flowering, five rapeseed plants were randomly selected for each of the following treatments: *control, pollinator exclusion* and *pollen addition*. The plants assigned for *pollinator exclusion* were bagged with a mesh bag (tulle, 65 × 40 cm, mesh size: 1 × 1 mm) before the onset of flowering and the bag stayed on the plant until harvest time. The plants assigned to the treatment *pollen addition* received extra pollen by hand-pollination besides the natural pollination. We visited the plants two times per week during the flowering period (between 17 September and 11 November) and hand-pollinated all open flowers, using a cotton swab on which pollen from a minimum of three different plants had been collected. New pollen was collected on a new cotton swab after we had pollinated approximately 10 stigmas. Finally, the control plants were available to insects during the full flowering period. No pesticides or fertilizers were applied at any time during the experiment.

### (c) Data collection

When the rapeseed was ripe, we counted the number of developed fruit capsules, also known as siliques, per plant. Ten randomly selected fruit capsules per plant were harvested and dried. The seeds from each individual fruit capsule were counted and weighed (lowest recorded weight with the scale was 0.5 mg). Some of the seeds were misshapen (6.2% of the seeds were ‘wrinkled’), some were attacked by fungus (5.6%) and some had started to germinate (0.15%). These misshapen, attacked and germinated seeds were counted as developed seeds but were not weighed. Out of the 345 plants (115 in each treatment) initially included in the experiment, 325 plants (104 pollinator excluded, 113 control and 108 pollen addition plants) were harvested, while the remaining plants were damaged by livestock or destroyed by other reasons. We estimated the difference in yield between treatments by calculating the mean number of seeds per plant for each treatment and site and then multiplying it with the mean seed weight for each specific site.

### (d) Local land use and floral survey

In a hectare plot surrounding the central house for each homegarden we schematically mapped all major land uses, including wooded land, shrub land, grazing land, perennial and annual crop land (table 1). Floral resources, which comprise flowering grasses, herbs, crops, shrubs and trees, were surveyed at the end of October 2012. An experienced surveyor surveyed the hectare for floral resources by slowly walking through each identified land-use polygon in the plot. This method was suitable for the patchy distribution of floral resources in the homegardens and ensured that all major floral resources were recorded. Frequently observed flowering species were: *Ageratum conyzoides*, *Guizotia schimperi*, *Persea americana*, *Rumex nepalensis*, *Achyranthes aspera* and *Bidens biternata*. The abundance of each flowering species was estimated on a logarithmic scale where 1 = 10–100 flowers, 2 = 101–1000 flowers and 3 > 1000 flowers. Floral units were defined depending on type of flower, e.g. for composite flowers the flower head was considered as one floral unit, whereas each flower was considered as one floral unit for plants with more distinct flowers. For total floral abundance, we translated each species abundance value to an estimated number of flowers (i.e. abundance category 1 = 50 flowers; 2 = 500 flowers and 3 = 5000 flowers) and summarized it for all flowering species per site. We defined floral richness as the total number of flowering plant species at each site.

### (e) Bee survey

Bees were surveyed in each site from August to October 2012 with two yellow vane traps and three pan traps: yellow, white and blue, respectively. The traps were filled with salty water as preservative (100 g salt l^−1^ water) and a drop of soap to reduce water surface tension. The traps were placed in the homegardens continuously for 86 ± 1 days from the beginning of August to the end of October, which largely overlaps with the period of rapeseed flowering. To reduce flooding of the pan traps, two holes were drilled close to the cup edge. The traps were emptied of insects and refilled with liquid two times per week in August and three times per week during the remaining part of the sampling period. All bees were collected and stored in alcohol for later identification. For further details on the bee survey, see [21].

### (f) Landscape classification

In a circle with a radius of 700 m surrounding each central house, we classified all pixels from a satellite image into either open, forest or other wooded areas. The program ChorosLandCover 0.9.0.2 (Izolde and Choros Cognition Company 2012) was used to classify the satellite image, which was a pan-sharpened high-resolution (0.5 m) big world view2 satellite image from October to November 2011, projected in UTM WGS 84. ChorosLandCover 0.9.0.2 provides an unsupervised classification method that takes into account the value of each separate pixel in addition to the values of surrounding pixels for the final classification. For detailed information on the classification process, see [21]. The surrounding classified area was divided into buffer bands, each 50 m in width, in which each land-use class was summed. As distant land uses probably have less impact on local pollinator communities and pollen limitation, we included weights (alpha) on the land-use variables to scale the impact of land use as a function of distance from the homegarden (equation (2.1)):
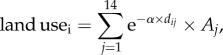
where *d_ij_* is the distance between the central house of homegarden *i* and the middle of the buffer band *j*, and *A_j_* is the total area of the land use of interest in each buffer band. We tested four weights (*α* = 0.01, 0.005, 0.001 and 0). The large alpha values results in a major impact of nearby land uses. By contrast, when alpha is set to zero the calculation equals the summing of the total land use within the 700 m (i.e. no weight) [21].

### (g) Statistical analyses

Data were averaged and analysed at the plant-level. We ran linear mixed models using the lme-function in package nlme in R 3.2.2 [29,30] to evaluate the effect of the three treatments (*control, pollinator exclusion* and *pollen addition*) on (i) the number of developed seeds per fruit capsules, (ii) seed weight (g), (iii) total number of developed fruit capsules per plant and (iv) total number of seeds per plant, which is the product of (i) and (iii). Site was included as a random factor. Total number of developed fruit capsules per plant and total number of seeds per plant were log_10_-transformed to meet the assumption of normally distributed residuals. For seed weight analysis, only weights of greater than 0.5 mg and a minimum of five seeds per fruit capsule were included. If there was a significant treatment effect, we calculated two predefined contrasts using the glht-function [31]. The predefined contrasts were between control and pollinator exclusion plants, to evaluate pollination services, and between pollen addition and control plants to evaluate pollen limitation.

We thereafter analysed total number of seeds per plant, as a proxy for yield, in relation to forest cover in the surrounding landscape, altitude, flower abundance and the area of annual crop within one hectare plot with site as random effect. As we were interested in pollen limitation, we omitted the pollination exclusion treatment in this analysis. Since forest cover variables were correlated, we first made eight separate models only including the fixed factors treatment and the interaction with either the *forest* or *wooded area* variable together with one of four weights (*α* = 0, 0.001, 0.005, 0.01) (following [21]). The forest cover variable included in the model with the lowest Akaike's information criterion (AIC) was regarded as the most appropriate forest cover variable and was selected for the full model. The variables floral abundance and floral richness were correlated (*r* = 0.58), and when comparing full models that either included floral abundance or richness, we found the model with floral abundance to provide the best fit (lowest AIC) and this term was therefore selected. Interaction terms between treatment and all other fixed factors were included in the full model. We simplified the models by doing likelihood-ratio tests between the full model and models with one of all the possible terms dropped. Models were fitted with maximum likelihood. The term with the highest *p*-value was dropped. The model was simplified until the model's AIC value no longer decreased with more deletions. The final model was refitted with restricted maximum likelihood [32]. We evaluated if local bee abundance and richness affected the pollen limitation of the plants by adding one of the following variables to the final model: abundance of bees including honeybees, abundance of bees without honeybees and bee species richness, and included their interaction with treatment. The bee variables can be considered to affect the pollen limitation only if the interaction with treatment was significant. We graphically evaluated all models by plotting the distribution of the residuals, the residual variation between treatments and the standardized residuals versus fitted values and versus each explanatory variable [32].

## 3. Results

### (a) Effect of treatment

**Figure 2.**
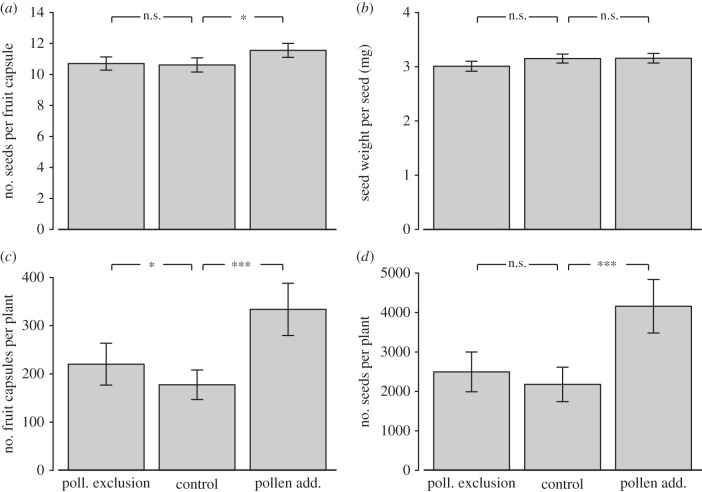
The rapeseed yield parameter means (±s.e.) for each treatment: pollinator exclusion, control, and pollen addition. (*a*) Number of seeds per fruit capsule, (*b*) seed weight per seed, (*c*) number of fruit capsules per plant, and (*d*) the total number of seeds per plant. The horizontal lines above the bars shows significance levels between treatments, where n.s., non-significant; *significant at the 5% level; ***significant at the 0.1% level.

The yield parameters seed set per fruit capsule (figure 2*a*; *F*_2,300_ = 3.1, *p* = 0.048), total number of developed fruit capsules per plant (figure 2*c*; *F*_2,300_ = 23.0, *p* < 0.001) and total seed set (figure 2*d*; *F*_2,300_ = 19.1, *p* < 0.001) were all affected by our pollination treatments (figure 2; electronic supplementary material, table S1). There was no difference in the mean weight of single seeds between treatments (figure 2*b*; *F*_2,296_ = 1.9, *p* = 0.15). The plants with pollen addition had on average one more seed per fruit capsule compared with the control plants (11.6 compared to 10.6 seeds, *p* = 0.042), while the plants where pollinators were excluded had the same amount of seeds as control plants (*p* = 0.95). The total number of developed fruit capsules per plant was on average 88% higher for the plants with pollen addition compared to the controls (*p* < 0.001), and 19% lower for the control plants compared with the plants with pollinators excluded (*p* = 0.034). The mean total seed set per plant varied considerably among sites (ranged between 226 and 8275 seeds for control plants). Plants with pollen addition produced on average 91% more seeds than control plants (*p* < 0.001), but we found no significant difference between controls and plants with pollinators excluded (*p* = 0.21). Overall, the mean estimated seed weight per plant was 13.0 g for the plants with pollen addition, 6.8 g for the control plants and 7.1 g for the plants with pollinators excluded.

### (b) Forest cover, bees and pollen limitation

We found no significant interaction effect between treatment (control and pollen addition) and our measured local and landscape variables on total seed production. Hence, the degree of pollen limitation (the difference between the seed set of control and pollen addition plants) was not connected to either forest cover, flower abundance, area of annual crops or altitude. After deletions of the interaction terms, the total seed production was affected by treatment (*t*_197_ = 6.6, *p* < 0.001) and by flower abundance (*t*_20_ = −2.2, *p* = 0.038; electronic supplementary material, table S2). Across the three-months sampling period, we collected a total of 484 bees, of which 170 were honeybees, in the pan and vane traps, with the average catch rate of 0.05 bees per day and trap. A total of 66 species were identified from five families; Apidae, Halictidae, Megachilidae, Colletidae and Andrenidae. Most individuals were from the genera *Patellapis*, *Lasioglossum*, *Lipotriches* and *Seladonia*, which are all from the Halictidae family*.* Variations in bee abundance and bee diversity among sites did not affect the degree of pollen limitation of the rapeseed plants among sites, as shown by the lack of significant interactions between treatment and abundance of bees, with (*t*_196_ = 0.58, *p* = 0.56) or without (*t*_196_ = 0.053, *p* = 0.96) honeybees included, and between treatment and bee diversity (*t*_196_ = −0.44, *p* = 0.66) (see the electronic supplementary material, table S3).

## 4. Discussion

We detected pollen limitation throughout our tropical agricultural study landscape. The number of fruit capsules and total seed set of the rapeseed plants increased greatly when plants had received extra pollen compared with control plants. However, there was no difference in total seed set between control and pollinator exclusion plants, which indicated a lack of pollination services. Contrary to our hypothesis, we found no effect of either the amount of surrounding forest or bee abundance or richness on the degree of pollen limitation. Thus, the pollination service is apparently inadequate for optimal yields of rapeseed across the whole landscape despite its heterogeneous composition and we suggest that this limitation may be linked to a low bee abundance in the focal landscape. The general consensus when low pollination services are detected in agricultural landscapes is that it is caused by a decrease in pollinator numbers, which is owing to, for example, intensification of agriculture or lack of natural habitats [33]. However, there might be no intrinsic ‘law’ that leads to high pollination success of crops in all heterogeneous mosaic agricultural landscapes. There may be landscapes with naturally low pollinator abundances that may be less suitable for widespread cultivation of crops in need of pollinators.

The low catch rates of bees in our vane and pan traps [21], compared with studies from the Northern Hemisphere with similar trapping methods (collecting more than two bees per day and trap compared with our catch rate of 0.05) (e.g. [34–36]), indicate low bee abundance in our focal landscape. The cause for the low bee abundance is yet unclear, but it seems not to be caused by high precipitation during our sampling since we had similar abundances in the same sites also in the dry season [21]. Neither are insecticides, which may decrease pollinator abundance and performance [9], widely used in our landscape. When we surveyed pollinators on coffee in 2011 in the same region, semi-wild honeybees were the completely dominating visitor with sometimes high abundances [22]. Two years later, both honeybees and other pollinators had low abundance in the coffee areas. That year the coffee started to bloom unusually early and the honeybee-keepers had not yet placed traditional beehives in the shade trees in the coffee areas. As a consequence, flowers stayed open for more than a week compared with 1–2 days 2 years before [22], and such delayed flower senescence is often an indication of inadequate pollination [37]. Irrespective of the mechanism, the observed low bee abundances and the high pollen limitation will have consequences for food production in the landscape.

The agricultural landscape in this area has plenty of floral resources, and the low management intensity, dominated by small-scale farming and homegardens, would suggest a good bee habitat. Owing to limited data on abundances of bees and other pollinators in sub-Saharan Africa, it is not possible to conclude whether our results are due to naturally low numbers of pollinators in these types of landscapes or if our landscape or sampling year is exceptional in some respect. In the few other manipulative pollination studies conducted in sub-Saharan agricultural landscapes, higher abundances and pollination services have been recorded [38–40]. For example, the fruit set of pigeon pea in eastern Kenya, on altitudes around 900–1000 m a.s.l., doubled or increased fourfold when accessible to pollinators compared with when pollinators were excluded [38,39]. Moreover, their bee catch rate per trap and day was approximately four times higher than in our study landscape (0.2 bees per trap and day; calculated from the electronic supplementary material, [38]). The relatively high elevation of our study area could potentially have influenced the low abundance of bees we found. Species richness and abundance of bees generally decline with altitude in both temperate and tropical systems and in both natural and disturbed habitats [41–44]. Lower abundance and richness of bees on higher altitudes may be linked to multi-level environmental filters like lower landscape diversity and local plant cover, but may also be affected more directly by abiotic factors like temperature leading to restricted energy consumption and lower foraging rates at higher altitudes [41,43,44]. Higher altitudes have also been found to affect the morphological traits of the bees, indicating the need for adaptations to higher altitudinal conditions [44,45]. Yet, abundances do not always decline with altitude since certain well-adapted species sometimes can reach high abundances in species poor high altitudinal settings as shown in a study from Mount Kilimanjaro, Tanzania [41]. Interestingly however, the abundance pattern on Mount Kilimanjaro, in relation to altitude, follows a U-shaped distribution with the lowest bee abundance on altitudes similar to the altitudes of our study (2000–3000 m versus 1900–2100 m a.s.l.) and with numbers comparable to ours (less than 0.1 bees per trap and day) [41]. Another possible factor influencing the bee abundance and the pollen limitation in the landscape is the presence of predators that beside direct top-down control may affect the behaviour and movement of the pollinators in the landscape, which can result in fewer and shorter flower visits [46,47]. Lack of nesting sites and material may also be a limiting factor for bee communities [48]. Even though there seems to be plenty of possible nesting opportunities in the landscape, interacting species like aggressive ants may hinder the use of them (cf. [49]). Studies that compare bee abundances in landscapes at different altitudes as well as examining local limiting factors for bees are necessary in order to come closer to an explanation to the found patterns in our study landscape.

Although treatment effects were consistent, we found a large variation in the rapeseed yield among sites. In contrast to our hypothesis, the variation in pollen limitation among sites was not related to the amount of forest in the surroundings. Even though we have found bee species richness and abundance to be positively affected by more forest in the surrounding area [21], this effect did not in turn result in higher yields of rapeseed. Perhaps this was owing to the fact that the relationship between bee species richness or abundance and forest cover actually were quite weak [21], but also that rapeseed are not solely pollinated by bees [50] and other pollinators might not follow the same gradients as bees. The forests in the region are constantly disturbed by anthropogenic activity such as coffee plantations, forest grazing, harvest for timber and firewood [51], which may lower the forests' quality as a habitat for bees. The variation in total seed set for both extra pollinated and control plants was weakly affected by the local abundance of flowers, with a lower seed set in areas with higher flower abundance. A possibility could be that higher abundance of flowering plants competed for and possibly diluted the pollinators on the local scale. However, if the effect of floral abundance was strong we should have seen a significant interaction between treatment and floral abundance, as control plants should have been more strongly affected by the competition compared with hand-pollinated plants. Therefore, these results should be interpreted with caution. The somewhat surprising result that the number of fruit capsules was higher for pollinator excluded compared with control plants could depend on the possible protection the bags provided for the plants against herbivores and pollen predators (cf. [52]). Cetoniidae beetles were common visitors of rapeseed flowers in the landscape (U. Samnegård 2012, personal observation) and may have reduced the potential yield for both control and pollen addition plants.

The benefit of pollination services to rapeseed varies among cultivars, from varieties with no or low yield increases from external pollination to considerable yield increases [53–57]. The rapeseed variety used in our experiment is clearly a variety that benefits from insect pollination. We found the differences in total seed production between control plants and plants with pollen addition to be mainly a consequence of the higher fruit set by pollen addition plants. Increased fruit set in rapeseed with insect pollination has been reported from several studies [53,54,58,59], as well as an effect on the number of seeds per fruit capsule [53,54,58,59] and seed weight [53,54,56,59] (but no effect in [58]), even though the effects vary with variety. The increase in fruit set with added pollination in our study was large (cf. [54]). The households could almost double their rapeseed yield if they could get sufficient pollination.

## 5. Conclusion

We found severe pollen limitation across a heterogeneous agricultural landscape in southwestern Ethiopia, which we suggest to be linked to the low bee abundance found in the landscape. The low bee abundance is somewhat surprising given that some of the identified threats to pollinators, like land-use intensification, pesticide application and lack of food resources and nesting sites, are less pronounced in our study landscape compared with many other areas with higher pollinator abundances. Some practices that commonly increase pollinator abundance, such as restricted use of pesticides and high availability of food and nesting resources, are already in place here, but are apparently insufficient. Thus, additional management strategies may be required to possibly enhance the pollination services in the landscape, to benefit the health and wealth of local farmers. Since the majority of pollination services to crops are typically provided by a small subset of the available pollinators [60], a first step may be to identify the main crop pollinators. Once these pollinators are identified, and their ecology is understood, appropriate strategies to enhance their abundance in the landscape can be developed. The presence of semi-wild honeybees are already more or less managed in the area by the provisioning of traditional beehives. Today the placement of beehives is mainly to optimize honey production. However, the beehives may also increase pollination services if placed in the vicinity of flowering homegarden crops. Beyond increasing pollination services in the landscape, yield gaps may be reduced by growing less pollinator-dependent varieties or by fertilizing the soils [53]. If the area of pollinator-dependent crops increases in the studied landscape, the problem with pollen limitation may be exacerbated unless targeted measures are taken.

## Supplementary Material

Statistical model structures and results

## Data accessibility

Datasets associated with this article are available at the Dryad Digital Repository: doi:10.5061/dryad.8rf3j [61].
