# Supplementary material for: A heterogeneous landscape does not guarantee high crop pollination
Source: Proc Biol Sci. 2016 Sep 14;283(1838):20161472. doi: 10.1098/rspb.2016.1472 (PMC5031663; doi:10.1098/rspb.2016.1472)
Supplement: Statistical model structures and results [file rspb20161472supp1.pdf]

Electronic supplementary data for the paper:

A heterogeneous landscape does not guarantee high crop pollination

Ulrika Samnegård, Peter A. Hambäck, Debissa Lemessa, Sileshi Nemomissa, Kristoffer Hylander

Proc. R. Soc. B 20161472. http://dx.doi.org/10.1098/rspb.2016.1472

Statistical model structures and results

Table S1. The results of linear mixed models analysing the effect of treatment (control, pollinator exclusion and pollen addition) on four different yield-components: seeds per fruit capsule, seed weight, total fruit set and total seed set.

response variable	*fixed effects*	*random stucture*	*F-value*	*numDf*	*denDF*	*p-value*
Seeds per fruit capsule	Treatment	Site	3.1	2	300	**0.048**
Seed weight (single seed)	Treatment	Site	1.92	2	296	0.15
Total fruit set per plant	Treatment	Site	23.0	2	300	**<0.001**
Total seed set per plant	Treatment	Site	19.1	2	300	**<0.001**

Table S2. Local and landscape factors effect on seed set. The full linear mixed model included the fixed factors: amount of surrounding forest cover, altitude, flower abundance and the area of annual crop within one hectare plot as well as their interactions with treatment. Only plant data from treatment *control* and *pollen addition* was included. Here we present the results from the final model after model simplification. No interaction terms remained in the final model.

response variable	*fixed effects final model*	*random structure*	*Df*	*t-value*	*p-value*
Total seed set per plant	Treatment	Site	197	21.7	<0.001
	Floral abundance		20	-2.4	0.028
	Annual crops and vegetables		20	-1.46	0.16

Table S3. The effect of bee diversity and abundance (with and without honeybees) on pollen limitation. Bee diversity and abundance was considered to affect the pollen limitation if a significant interaction effect was found between the bee-variable and treatment. Bee variables were added to the final linear mixed model (Table S2). Component factors of the interactions are included in the model but only the results from the interactions are presented.

response variable	*fixed effects final model +*	*random structure*	*Df*	*t-value*	*p-value*
Total seed set per plant	Treatment x Bee diversity	Site	196	-0.44	0.66
Total seed set per plant	Treatment x Bee abundance (incl. Honeybees)	Site	196	0.63	0.53
Total seed set per plant	Treatment x Bee abundance (excl. Honey bees)	Site	196	0.05	0.96
